# DNA barcoding and morphology reveal two common species in one: Pimpla molesta stat. rev. separated from *P. croceipes* (Hymenoptera, Ichneumonidae)

**DOI:** 10.3897/zookeys.124.1780

**Published:** 2011-08-18

**Authors:** Anu Veijalainen, Gavin R. Broad, Niklas Wahlberg, John T. Longino, Ilari E. Sääksjärvi

**Affiliations:** 1 Department of Biology, FI-20014 University of Turku, Turku, Finland; 2 Natural History Museum, Cromwell Road, London SW7 5BD, UK; 3 Lab 1, Evergreen State College, Olympia, Washington, 98505, USA

**Keywords:** cryptic species, integrative taxonomy, Neotropics, parasitoid wasp, Pimplinae

## Abstract

Correct species identification is the basis of ecological studies. Nevertheless, morphological examination alone may not be enough to tell species apart. Here, our integrated molecular and morphological studies demonstrate that the relatively widespread and common neotropical parasitoid wasp *Pimpla croceipes* Cresson, 1874 (Hymenoptera: Ichneumonidae: Pimplinae) actually consists of two distinct species. The name *Pimpla molesta* (Smith, 1879), stat. rev. is available for the second species. The two species were identified by DNA barcoding and minor differences in morphology and colouration. Our results support the previous notions that DNA barcoding can complement morphological identification and aid the discovery of cryptic species complexes.

## Introduction

In the midst of global biodiversity loss and substantial taxonomic shortcomings, improved identification methods for hyperdiverse and poorly known invertebrate groups are good news. Integrating molecular methods with morphological species identification can accelerate biodiversity inventories and facilitate spotting cryptic species (i.e. two or more distinct species classified as one due to morphological similarity; e.g. Smith et al. 2009). Since ecological research ultimately depends on the work of taxonomists, consistent species definitions are important for attaining reliable scientific results. In this paper, we use DNA barcoding to reveal a cryptic complex masked within a common and widespread neotropical parasitoid wasp species.

**Figure 1.**
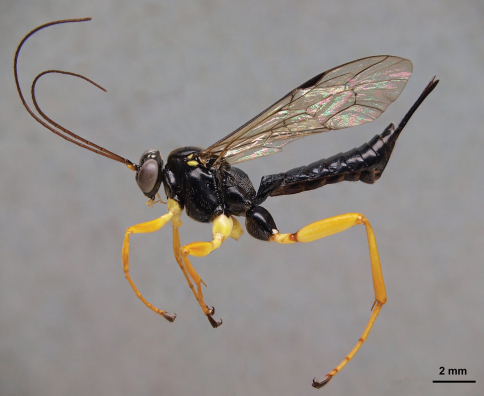
*Pimpla croceipes* female, lateral view.

**Figure 2.**
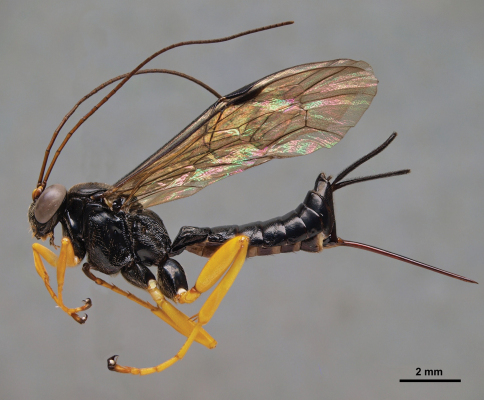
*Pimpla molesta* female, lateral view.

The parasitoid wasp family Ichneumonidae (Hymenoptera) may well be the largest animal family on earth, but it is considered taxonomically challenging and poorly known (Gauld 2000). The vast majority of ichneumonids are parasitoids; they lay their eggs in or on other arthropods and the developing parasitoid eventually kills the host. The tropical ichneumonid fauna is generally undersampled, although Ian Gauld’s tremendous work on the Costa Rican ichneumonids yielded many practical keys for identifying Central American species (Gauld 1991, 1997, 2000, Gauld et al. 1998, 2002a,b). Most neotropical ichneumonids are either rare or rarely collected, as even extensive sampling produces only a few specimens of most species (Sääksjärvi et al. 2004). However, as in any fauna, there are still some commonly encountered neotropical ichneumonids, such as the characteristic pimpline *Pimpla croceipes* Cresson, 1874 (Fig. 1), whose distribution has been reported to extend from Mexico through Central America to Colombia, Ecuador and Venezuela (Yu et al. 2005). With its wide distribution, it is not surprising that there has been a junior synonym of *Pimpla croceipes* recognised, namely *Pimpla molesta* Smith, 1879 (Fig. 2), synonymized with *Pimpla croceipes* by Cameron (1886) (see Gauld 1991).

When working through neotropical ichneumonid samples, we encountered a number of specimens that we identified as *Pimpla croceipes* (hereafter *Pimpla croceipes* sensu lato) according to the keys in Gauld (1991) and Gauld et al. (1998), but which showed some variation in colouration and sculpture. This variation could not be readily compartmentalised as distinct species but this led us to hypothesize that the specimens represented a complex of species morphologically close to one another. As integrating DNA barcoding with other species identification methods has proven an efficient method for separating cryptic ichneumonoid species complexes (Janzen et al. 2009, Smith et al. 2009), we tested our hypothesis by comparing the results of DNA barcoding and careful morphological examination.

## Methods

The data consisted of specimens collected by the LLAMA project in Guatemala and Honduras (see below), currently on loan to the Zoological Museum, University of Turku (ZMUT) and later to be deposited in the collaborative institutions of the LLAMA project, and the collections of the Natural History Museum, London, UK (BMNH). The LLAMA specimens were studied using both molecular and morphological species identification methods, the BMNH specimens focusing exclusively on morphology. The images were taken in ZMUT using an Olympus SZX16 stereomicroscope attached to an Olympus E520 digital camera. The layer photos were combined using the programmes Deep Focus 3.1, Quick PHOTO CAMERA 2.3 and Combine ZP.

### LLAMA

**Figure 3.**
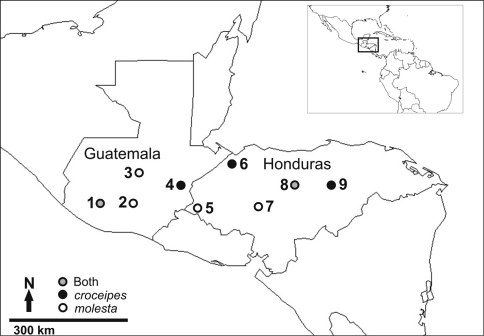
The LLAMA study sites where specimens of either *Pimpla croceipes*, *Pimpla molesta*, or both, were collected **1** Suchitepequez **2** Sacatepequez **3** Baja Vera Paz **4** Zacapa **5** Ocotepeque **6** Cortés **7** Comayagua **8** Olancho: “La Muralla” **9** Olancho: “Catacamas”. See text for more specific descriptions of site locations.

In the LLAMA samples, there were 97 specimens of *Pimpla croceipes* s.l. The specimens were collected by Malaise traps as a part of the Leaf Litter Arthropods of Mesoamerica project (LLAMA; http://llama.evergreen.edu) led by JTL in Guatemala and Honduras from May to June 2009 and 2010, respectively (see online Supplementary material for geospatial information and sampling periods). The LLAMA project applied similar Malaise sampling effort at 17 study sites, ranging from 50–2400 m asl. Four or five Malaise traps were set for four days at each site. *Pimpla croceipes* s.l. specimens were collected at nine of the 17 sites (Suchitepequez: 4 km S volcano Atitlán, Sacatepequez: 5 km SE Antigua, Baja Vera Paz: Biotopo El Quetzal, Zacapa: 2 km SE La Unión, Ocotepeque: 13 km E Nueva Ocotepeque, Cortés: Parque Nacional Cusuco, Comayagua: 10 km E Comayagua, Olancho: Parque Nacional La Muralla, and Olancho: 9–11 km N Catacamas; Figs 3, 5). All nine sites were at mid to high elevation, 1200–2335 m asl. Habitats were all mature wet forest, typically diverse mesophyll cloud forest, but some sites with variable densities of pine, oak, and *Liquidambar*. Traps were generally located on forest edges or in small clearings. Sampling took place during the transition from dry season to wet season.

### DNA barcoding

We extracted the DNA of all the LLAMA specimens of *Pimpla croceipes* s.l.using the DNeasy® Blood & Tissue Kit (QIAGEN) and following the standard bench protocol for animal tissue in DNeasy Blood & Tissue Handbook 07/2006 (the samples were also incubated at 70° C for 10 minutes after adding the Buffer AL and vortexing). Next, we amplified the approximately 650-base fragment of the 5’ end of the mitochondrial gene cytochrome *c* oxidase I(*cox1*, COI) well known as the barcode region for animals. Each PCR was done in a 20 µl volume consisting of 1 µl of DNA extract and 19 µl of master mix (12.5 µl dH2O, 2.0 µl 10× PCR Gold Buffer, 2.0 µl MgCl2 solution, 1.0 µl primer LCO, 1.0 µl primer HCO, 0.4 µl dNTP, 0.1 µl Ampli Taq Gold). The PCRs were run for 40 cycles with an annealing temperature of 50°C. The succesful PCRs were cleaned and sequenced by Macrogen (South Korea), after which we edited and aligned the sequences and constructed the neighbour-joining tree based on genetic distance calculated with the K2P model using MEGA v4 (Kumar et al. 2004). The LLAMA specimens for which we were not able to extract DNA or receive readable sequences were still included in the morphological studies. Sequences have been deposited in GenBank under accession numbers JN387917–JN387993. Specimens of two species of *Pimpla* from GenBank (*Pimpla aequalis* ProvancherAF146681 and *Pimpla* sp. FN662469) were used as outgroups for the neighbour-joining analyses. Support was assessed using bootstrapping with 1000 pseudoreplicates.

### BMNH collection

In the Natural History Museum, there were 237 Costa Rican specimens, 24 Mexican specimens, 2 from Panama and 1 from Venezuela, plus the holotype of *Pimpla molesta*, from Costa Rica (Supplementary material). These were sorted into *Pimpla croceipes* and *Pimpla molesta* based on their morphological characters (Table 1).

**Table 1.** A comparison of the diagnostic morphological characters of *Pimpla croceipes* and *Pimpla molesta*.

Sex	Character	*Pimpla croceipes* (Fig. 1)	*Pimpla molesta* (Fig. 2)
F	Subalar prominence	Yellow/white	Black
F	Fore coxa	Yellow	Black or black and yellow
F	2nd tergite	More closely punctate(Fig. 6A)	More sparsely punctate (Fig. 6B)
M	2nd tergite	Transverse groove at or behind posterior 0.65 (Fig. 6C), curved; tergite usually more closely punctate	Transverse groove at posterior 0.55 (Fig. 6D), almost straight; tergite more sparsely punctate

## Results

**Figure 4.**
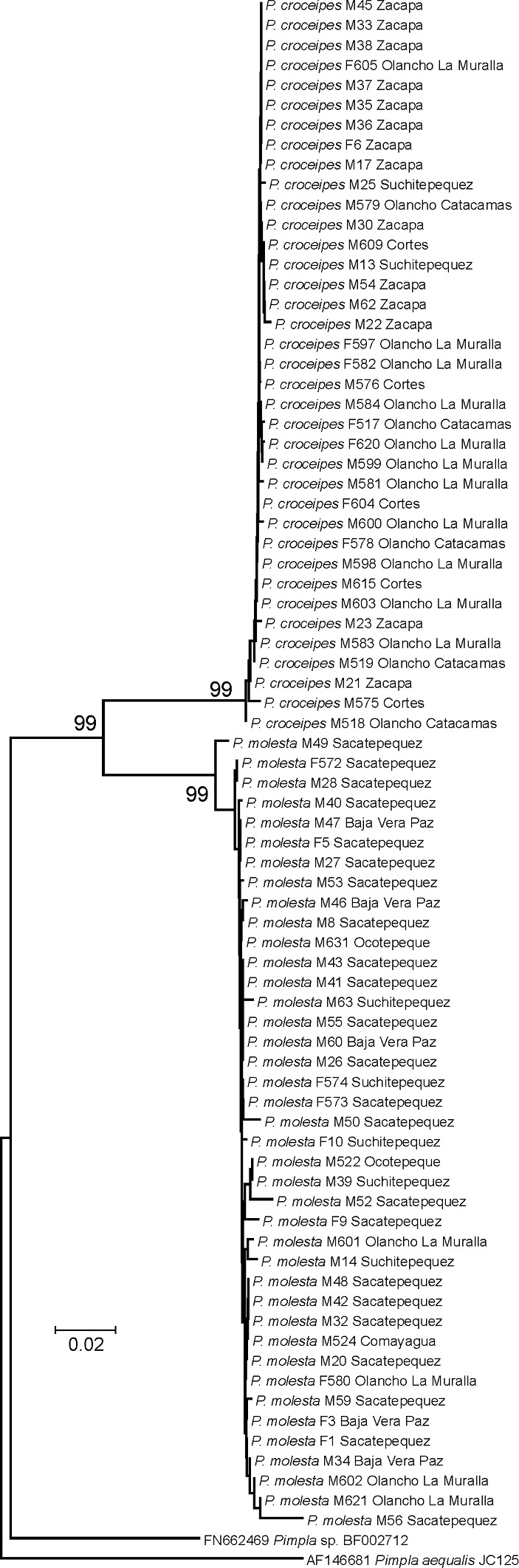
Neighbour-joining tree of the successfully DNA barcoded LLAMA specimens (species; sex; DNA voucher code; location). Numbers above the branches are bootstrap proportions. The two species *Pimpla croceipes* and *Pimpla molesta* (stat. rev.) are clearly separated into two well-supported clusters. The results were further supported by morphological examination.

We obtained COI sequences for 77 specimens and the DNA barcoding results clearly group the specimens into two distinct species (Fig. 4). The two species differed from each other by 9.9% (K2P distance), and had virtually no intraspecific variation (0–0.5% variation). The molecular results, enabling us to preliminarily divide the successfully barcoded LLAMA specimens into two groups, greatly facilitated the morphological identification process, and we also found interspecific differences in the specimens’ colouration and morphology (Table 1). Careful morphological examination and comparison with type material revealed that the two species each have available names: *Pimpla croceipes* and *Pimpla molesta* (stat. rev.). The ecological information that was available (e.g. altitude, habitat) could not be used to entirely predict the identity of the specimens as the distribution areas of the two species overlap in the total LLAMA dataset and both species could occur in the same traps (Supplementary material). This is in accordance with Gauld et al. (1998) who noted that many common Central American species of *Pimpla*, namely *Pimpla croceipes* s.l., *Pimpla croceiventris* (Cresson) and *Pimpla sedula* Cameron, occur sympatrically.

We examined the holotype female of *Pimpla molesta* in BMNH and images of the lectotype female of *Pimpla croceipes* (deposited in Philadelphia Academy of Sciences). Fortuitously, these two types correspond to our two morphotypes, designated on the basis of the molecular separation. We were therefore able to assign all of our specimens of *Pimpla croceipes* s.l.to either *Pimpla croceipes* or *Pimpla molesta*. The species can be identified according to the diagnosis below and separated with the character differences summarized in Table 1 and illustrated in Figures 1, 2 and 6.

Males are relatively straightforward to separate on the basis of the sculpture on the second metasomal tergite, although colour pattern appears to offer no differences. Females are more difficult to separate but do show small differences in colour and metasomal sculpture. Table 1 should serve to separate almost all individuals. Both key easily to *Pimpla croceipes* using Gauld’s (1991) and Gauld et al.’s (1998) keys to Costa Rican species and Gauld et al.’s (2002b) key to El Salvadorean species. Large females of *Pimpla molesta* (fore wing length ~12mm) may have a partly yellow/white subalar prominence and only a small amount of black on the fore coxa. However, the subalar prominence is not entirely yellow or white which, coupled with the difference in sculpture of the second tergite, should allow separation of all individuals. Conversely, some small females of *Pimpla croceipes* (fore wing length ~8.5mm) have the fore coxa extensively dark marked and the subalar prominence a rather dull cream, but the subalar prominence is nevertheless entirely pale-marked and the sculpture differences still stand. Interestingly, the two *Pimpla* species seem to share a mimicry pattern with a *Lissonota* species (Ichneumonidae: Banchinae), represented by two males collected by Malaise trap in another sample at the Honduran site Comayagua and illustrated in Figure 7 (LLAMA sample code Ma-C-04-1-02). This is in accordance with the notion of Gauld et al. (2002a) that many of the Costa Rican montane *Lissonota* are involved in mimicry complexes characterized by black colouration with lower body parts being bright yellow.

In total, we studied 361 specimens (excluding the holotypes) and finally assigned them into 175 individuals of *Pimpla croceipes* and 186 of *Pimpla molesta* (Supplementary material). The two species were equally abundant in the LLAMA samples (*Pimpla croceipes*: 48, *Pimpla molesta*: 49 individuals). In BMNH, there are *Pimpla croceipes* specimens from Costa Rica and Venezuela (Las Mercedes) and *Pimpla molesta* specimens from Costa Rica, Mexico (several sites in Guerrero State) and Panama (Chiriquí).

**Figure 5.**
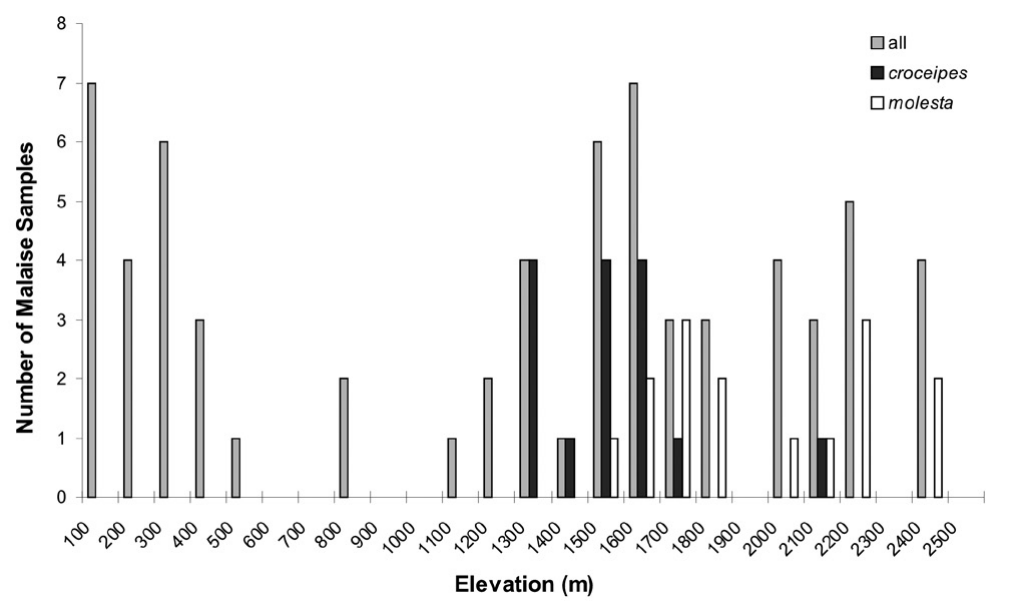
A frequency histogram of elevations of Malaise samples, for **1)** all LLAMA samples, **2)** those with *Pimpla croceipes*, and **3)** those with *Pimpla molesta*.

## Discussion

We confirm with morphological and molecular evidence and occurrence of sympatry that *Pimpla croceipes* and *Pimpla molesta* are two separate species. Gauld (1991) observed similar morphological variation as that reported here but, in the absence of molecular data, he concluded that these differences represented intraspecific variation among *Pimpla croceipes* rather than interspecific variation between two species. He wrote (referring to *Pimpla molesta* stat. rev., based on specimens in BMNH): “Variation: Small individuals tend to have the metasomal tergites very sparsely and weakly punctate; often such specimens lack black marks on the tegula and subalar prominence and a few individuals even have the scutellum black. Specimens from the wettest sites also often lack the yellow mark on the subalar prominence.”

When cryptic species are discovered, linking previously compiled species data to the correct “new” species may be difficult (Schlick-Steiner et al. 2007). Gauld (1991) reports that in Costa Rica, the species (*Pimpla croceipes* s.l.) is very common in humid areas at 800–1600 m asl where it comprises 7–35% of pimpline individuals in Malaise trap samples. According to our observations, both species are very common in Central American cloud forests: in the Guatemalan and Honduran LLAMA Malaise trap samples studied here, *Pimpla croceipes* accounts for 22.2% and *Pimpla molesta* for 22.7% of all the pimplines collected (50–2400 m asl). When the trap sites are plotted along an altitudinal gradient, there is a degree of altitudinal separation between the two species, with *Pimpla croceipes* ranging from 1210 to 2010 m and *Pimpla molesta* at somewhat higher altitudes, from 1480 to 2335 m (Fig. 5, Supplementary material). Costa Rican specimens in BMNH show a fairly clear altitudinal separation, with no specimens of *Pimpla croceipes* having been collected above 1300 m, whilst 60% of the *Pimpla molesta* specimens were collected above 1300 m (Supplementary material). All of the Mexican specimens in BMNH were collected at relatively high altitude (1800–2900 m) and all proved to be *Pimpla molesta*. The species’ distribution ranges overlap as they were collected in the same LLAMA samples, yet the species were at their most abundant at different LLAMA sites; 19 out of 48 specimens (40%) of *Pimpla croceipes* were collected at Zacapa, and 23 of 49 specimens (47%) of *Pimpla molesta* at Sacatepequez. Both of the species were found in cloud forests at mid-elevations (approx. 1500–1750 m asl) (Fig. 5, Supplementary material). The biology of the species is not known, and we are not aware of any rearing records for them either. In fact, there are only very few host records for tropical American species of *Pimpla*, which may be due to their biology (see Díaz 2000, Gauld et al. 2002b). In other parts of the world, *Pimpla* species are known to be idiobiont endoparasitoids of lepidopteran pupae in concealed locations (e.g. in soil, leaf litter or leaf rolls).

We identify at least three sources of error that should be kept in mind while interpreting the presence of the two *Pimpla* species in specific study localities. First, the sampling efficiency of Malaise traps may be influenced by the precise positioning of the trap in the sampling locality. Second, many species of ichneumonids are often rare in samples. For this reason, their presence or absence in a Malaise trap sample may be largely a coincidence. Third, pimplines are large parasitoids which are normally strong fliers. Thus, a Malaise trap may sample individuals that are just passing a forest patch instead of actually being resident there.

We have shown that the *Pimpla croceipes* s.l., previously thought to be one species, is in fact two morphologically very similar but molecularly clearly different species which are both common and co-occur in Central America. As with other studies on neotropical parasitoids (Smith et al. 2007, 2008, Janzen et al. 2009), we found DNA barcoding to complement morphological identification and to aid the discovery of cryptic species complexes. Integrative taxonomy studies continually find cryptic species both from the temperate and tropical regions (Bickford et al. 2006). Whether cryptic parasitoid species in the tropics are prevalent enough to significantly raise the estimations of the total number of parasitoid species remains to be seen.

**Figure 6 (A–D).**
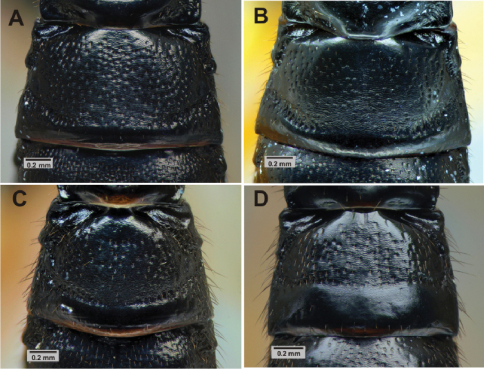
Metasoma. 2nd tergite sculpture of *Pimpla croceipes* female **A,** *Pimpla molesta* female **B,** *Pimpla croceipes* male **C,** and *Pimpla molesta* male **D**.

**Figure 7.**
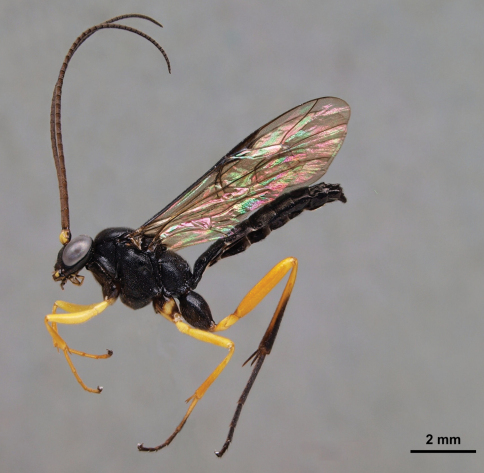
*Lissonota* sp. male (Ichneumonidae: Banchinae) collected at Comayagua (Honduras) showing a similar mimicry pattern to *Pimpla croceipes* and *Pimpla molesta*.
